# Investigation of candidate genes involved in the rhodoquinone biosynthetic pathway in *Rhodospirillum rubrum*

**DOI:** 10.1371/journal.pone.0217281

**Published:** 2019-05-21

**Authors:** Amanda R. M. Campbell, Benjamin R. Titus, Madeline R. Kuenzi, Fernando Rodriguez-Perez, Alysha D. L. Brunsch, Monica M. Schroll, Matthew C. Owen, Jeff D. Cronk, Kirk R. Anders, Jennifer N. Shepherd

**Affiliations:** 1 Department of Chemistry and Biochemistry, Gonzaga University, Spokane, Washington, United States of America; 2 Department of Biology, Gonzaga University, Spokane, Washington, United States of America; University of Alberta, CANADA

## Abstract

The lipophilic electron-transport cofactor rhodoquinone (RQ) facilitates anaerobic metabolism in a variety of bacteria and selected eukaryotic organisms in hypoxic environments. We have shown that an intact *rquA* gene in *Rhodospirillum rubrum* is required for RQ production and efficient growth of the bacterium under anoxic conditions. While the explicit details of RQ biosynthesis have yet to be fully delineated, ubiquinone (Q) is a required precursor to RQ in *R*. *rubrum*, and the RquA gene product is homologous to a class I methyltransferase. In order to identify any additional requirements for RQ biosynthesis or factors influencing RQ production in *R*. *rubrum*, we performed transcriptome analysis to identify differentially expressed genes in anoxic, illuminated *R*. *rubrum* cultures, compared with those aerobically grown in the dark. To further select target genes, we employed a bioinformatics approach to assess the likelihood that a given differentially expressed gene under anoxic conditions may also have a direct role in RQ production or regulation of its levels *in vivo*. Having thus compiled a list of candidate genes, nine were chosen for further study by generation of knockout strains. RQ and Q levels were quantified using liquid chromatography-mass spectrometry, and *rquA* gene expression was measured using the real-time quantitative polymerase chain reaction. In one case, Q and RQ levels were decreased relative to wild type; in another case, the opposite effect was observed. These results comport with the crucial roles of *rquA* and Q in RQ biosynthesis, and reveal the existence of potential modulators of RQ levels in *R*. *rubrum*.

## Introduction

**Fig 1:**
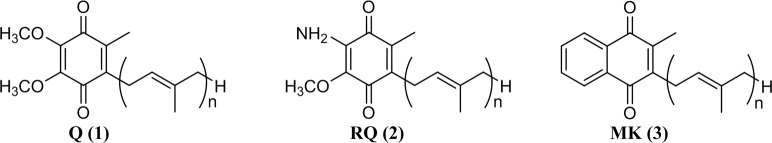
Structures of ubiquinone, rhodoquinone and menaquinone. The number of isoprene units (n) in the tail varies by species from 6–10. The reduction potentials of the quinones are as follows: Q, *E*°′ = +100 mV; RQ, *E*°′ = −63 mV; MK, *E*°′ = −80 mV.

Bacteria and simple eukaryotic organisms that have adapted to anoxic or hypoxic conditions for all or part of their life-cycle employ a variety of metabolic strategies to cope with such environments [1,2]. One such strategy relies upon fumarate (*E*°′ = +30 mV) as an electron acceptor in a reversal of the succinate dehydrogenase (SDH) reaction of the citric acid cycle that comprises a fundamental component of aerobic metabolism. While ubiquinone (Coenzyme Q or Q, Fig 1, compound 1) is the electron acceptor in the SDH reaction, a quinone with a lower standard reduction potential is required to make fumarate reduction more favorable. Rhodoquinone (RQ) (Fig 1, compound 2) or menaquinone (MK) (Fig 1, compound 3) are naturally occurring compounds that meet this requirement [3].

The SDH reaction is catalyzed by the Complex II family of integral membrane, multisubunit enzymes. Two homologous forms of Complex II are recognized and characterized: succinate:ubiquinone reductase (SQR) and quinol:fumarate reductase (QFR), that are optimized to function in aerobic and anaerobic metabolism, respectively. In *E*. *coli*, the expression of these two Complex II homologs is adjusted in response to oxygen levels, with hypoxic and anoxic conditions promoting QFR predominance, along with a shift in composition of the quinone pool toward MK with which QFR functions most efficiently [4]. The change in Complex II homolog expression and the adjustment to a lower potential quinone is crucial to the thermodynamic and kinetic favorability of fumarate reduction. A similar scenario is played out in the parasitic nematode, *Ascaris suum*, when migration into a host confronts the organism with lowered oxygen levels. In response, the subunit composition of Complex II is altered in favor of fumarate reduction; in this case, RQ is employed as the low potential electron carrier, and it becomes the predominant mitochondrial quinone component [5].

Since the discovery of RQ in the alphaproteobacterium *Rhodospirillum rubrum* [6], RQ has only been found in a limited number of bacterial and eukaryotic species. Although it fulfills an analogous role, RQ stands in marked contrast to MK in structure, biosynthesis, phylogenetic distribution and evolutionary origins [3]. The occurrence of MK, a naphthoquinone, among prokaryotic organisms is extremely broad, more so than Q (confined to alpha-, beta-, and gamma-proteobacteria), reflecting its earlier evolutionary origins in an anoxic environment. The much more restricted phylogenetic distribution of RQ, which like Q is a benzoquinone, points to a relatively recent origin of RQ from an augmentation of Q biosynthesis, possibly even later than the divergence of the eukaryotic lineage [7].

The study of RQ in *R*. *rubrum* has revealed further details about its biosynthetic origins and relationship to Q. As a photosynthetic facultative anaerobe, *R*. *rubrum* grows aerobically, utilizing Q in the dark, but also photoheterotrophically with light under anaerobic conditions. While in the latter case, *R*. *rubrum* would be expected to utilize RQ and QFR (as is the case in parasitic helminths and a few other eukaryotic species that have adapted to anoxic environments) [2,8], a recent study by Ghosh, et al. supports the conclusion that *R*. *rubrum* lacks an orthologous QFR, implying that SQR is capable of functioning in reverse with RQ as an electron donor [9]. A similar phenomenon has been reported in *E*. *coli*, where SQR can replace QFR to support anaerobic growth when fumarate is utilized as the terminal electron acceptor using the low potential carrier, MK [10]. We previously reported that Q is a required precursor for RQ biosynthesis in *R*. *rubrum* [11]. Investigation of a mutant strain incapable of anaerobic growth and devoid of RQ led to the identification of the *rquA* gene as the locus of this loss of function. Complementation of the mutant with an intact *rquA* gene restored both anaerobic growth and RQ production [12].

The phylogenetic distribution of *rquA* was analyzed in a recent report [13]. In addition to its sparse distribution across eukaryotes and bacteria adapted to hypoxia, this analysis supports the hypothesis that this pattern resulted from multiple lateral gene transfer events. Interestingly, this work highlights the fact that a subset of eukaryotic organisms (such as *A*. *suum* and *Caenorhabditis elegans*) known to produce RQ lack an *rquA* ortholog, suggesting that there are alternative biosynthetic routes to RQ. Nonetheless, it is clear that *rquA* performs a necessary role in *R*. *rubrum* (and presumably in all other organisms possessing *rquA* orthologs), a role we seek to further clarify in the present study. We report here the first transcriptome data obtained by RNA sequencing (RNAseq) of *R*. *rubrum* under aerobic and anaerobic conditions. This data, in conjunction with comparative genomic analysis, was used to evaluate putative gene candidates involved in RQ biosynthesis or its regulation. We then characterized several such candidates by generating knockouts in *R*. *rubrum* and assessing the effect on RQ levels and *rquA* expression.

## Materials and methods

### Bacterial strains and growth

Wild type *R*. *rubrum* (ATCC 11170) was obtained from ATCC (Manassas, VA). All *R*. *rubrum* cultures were grown in yeast extract-supplemented malate-ammonium rich (SMN) medium [12] supplemented with nalidixic acid (Nx, 20 μg/mL). Gentamicin sulfate (Gm, 10 μg/mL) was additionally added for all mutant strains. *R*. *rubrum* starter cultures (6 mL) were inoculated from a colony scrape on an SMN-agar plate, and grown aerobically in 25-mL Erlenmeyer flasks at 30°C in the dark in a C24 Incubator Shaker (New Brunswick Scientific, Edison, NJ), shaking at 150 rpm for 2 days (OD_660_ values from single colony ranged from 1–4). For growth experiments, aerobic and anaerobic cultures from each strain were prepared from the same seed culture which was diluted with SMN to a final OD_660_ of 0.02, and divided into replicate flasks or vials, respectively. Aerobic cells were grown using the same conditions described for starter cultures and harvested in the late stationary phase after 2.5 days (OD_660_ 4–5), which corresponded to maximum cell density and a semi-aerobic state (indicated by pink pigmentation of culture). Anaerobic *R*. *rubrum* cultures were grown in glass screw-top 5/8 dram vials filled to capacity at 30°C in an Innova 4430 incubator shaker (New Brunswick Scientific, Edison, NJ) equipped with a full spectrum fluorescence lamp with light output of 365 lx (Verilux Full Spectrum F20T12VLX, Veriflux, Inc., Waitsfield, VT). Anaerobic growth lagged about two days behind aerobic growth, and anaerobic cells were harvested in the late stationary phase after 5 days (OD_660_ ~3–8), which corresponded to deep burgundy pigmentation of cultures. Cultures were pelleted in microcentrifuge tubes (1.5 mL aliquots) by centrifugation at 10,000 x *g* for 20 min. After discarding supernatant, tubes were inverted on paper towels for 10 min to drain residual water, and then any remaining moisture on the inside of the tubes was removed using Kimwipes prior to obtaining final pellet masses (25–40 mg). Pellets from the same starting cultures of each strain were used for both LC-MS and RT-qPCR analysis. *E*. *coli* cells were grown overnight at 37°C in Luria broth while shaking at 180 rpm (OD_600_ of 2–3).

### RNA sequencing (RNAseq)

Wild type *R*. *rubrum* (ATCC11170) was grown from 5^th^ generation freezer stocks to the early stationary phase either aerobically or anaerobically as described previously [11], to create a 6^th^ generation of cultures with cell densities of 2 or 4 OD, respectively. The cells were pelleted by centrifugation (10,000 x *g)* and total RNA was extracted using the RNeasy Mini Kit (Qiagen, Palo Alto, CA). RNA sequencing was performed by the High Throughput Genomics Center (HTGC) at University of Washington in Seattle, WA. Sample integrity was tested using a bioanalyzer and RNA deep sequencing for prokaryotic organisms was performed using Illumina Single End Sequencing with 30–40 M reads at 1x36 base pair. Reads per kilobase million (RPKM) for every gene in the *R*. *rubrum* genome was computed as a measure of RNA quantity, and genes were sorted by comparing the ratio of RPKM in anaerobic versus aerobic conditions. Data were deposited in the Gene Expression Omnibus (GEO accession number: GSE130635) and are summarized in S1 Table.

### Genome comparisons

To identify likely orthologs among the genomes of *R*. *rubrum* (NCBI Genome ID 1016), *Rhodoferax ferrireducens* (NCBI Genome ID 1337), and *Rhodobacter sphaeroides* (NCBI Genome ID 509), each gene from the three genomes was mapped to orthologous gene groups among proteobacteria at the eggNOG database using the online tool eggNOG-mapper [14]. Subsequently, the gene groups were sorted to find groups shared by *R*. *rubrum* and *R*. *ferrireducens*, but not by *R*. *sphaeroides*. The database mapped 3398 *R*. *rubrum* (ATCC 11170) genes into orthologous gene groups. Of these, 1206 genes were mapped to orthologous groups shared by both *R*. *ferrireducens* T118 and *R*. *sphaeroides* 2.4.1, and an additional 269 *R*. *rubrum* genes were mapped to gene groups shared only with *R*. *ferrireducens*, but not with *R*. *sphaeroides* (S1 Table). A second approach was employed to measure the similarity of each *R*. *rubrum* gene to its closest relative in each of the other genomes in order to identify *R*. *rubrum* genes that are more closely related to *R*. *ferrireducens* genes than to *R*. *sphaeroides* genes. Each *R*. *rubrum* gene was aligned with its nearest relative in *R*. *ferrireducens* and *R*. *sphaeroides* by BLASTp and the strength of the alignment was recorded as an E-value. *R*. *rubrum* genes were sorted by the size of the distance between *R*. *ferrireducens* and *R*. *sphaeroides* using the ratio (E of *R*. *rubrum*:*R*. *ferrireducens* / E of *R*. *rubrum*:*R*. *sphaeroides*), where genes of interest had a very small E in a *R*. *ferrireducens* alignment and a much larger E in a *R*. *sphaeroides* alignment. Ratio values closest to zero indicated genes that are much more closely related to *R*. *ferrireducens* than to *R*. *sphaeroides*.

### Construction of *R*. *rubrum* knockout mutants

Two 0.8 kb fragments in the 5’ and 3’ regions of the gene of interest were separately amplified by high-fidelity PCR from WT *R*. *rubrum* genomic DNA using oligonucleotide primers with 5’ tails containing restriction sites. Primers constructed to make a precise deletion of the open reading frame were designed on the New England BioLabs website using the NEBuilder Assembly Tool and are listed in Table 1. A gentamicin antibiotic resistance (Gm^r^) cassette was amplified from the plasmid pUCGM (gift from Gary Roberts, University of Wisconsin, Madison) using primers with sequences (forward: 5’-CCATTCGCCATTCAGGCTG-3’ and reverse: 5’-GCAGTGAGCGCAACGCAATTAATG-3’) with added restriction sites corresponding to the 5’ and 3’ amplicons (Table 1). The amplicons were ligated together into the pUX19 plasmid (gift from Gary Roberts, Addgene plasmid # 36328), a kanamycin-resistant (Km^r^) suicide vector, using the Gibson Assembly kit (New England BioLabs, Ipswich, MA) and transformed into DH5α *E*. *coli* for replication. The isolated plasmid sequences were verified using Sanger sequencing, and S17-1 *E*. *coli* was transformed with each plasmid for subsequent conjugation with WT *R*. *rubrum*. Transformed S17-1 cultures were grown overnight to an OD_600_ of 2.0, diluted 1:4 in LB, and incubated at 37°C with shaking at 180 rpm for 2.5 h. Aerobically grown *R*. *rubrum* (100 μL, OD_660_ 2.0) was combined with 100 μL of the S17-1 culture and pelleted at 8000 *x g* for 30 s. The pellet was resuspended in 25 μL SMN media and added directly to a 0.45 μm x 15 mm nitrocellulose filter, which was placed on an SMN plate with no antibiotics and incubated overnight at 30°C. After 12–16 h, the filter was transferred to 1 mL SMN and gently agitated using a vortexer to resuspend cells. From this resuspension, 150 μL was plated on SMN plates containing Nx and Gm. Colonies that grew on the Nx/Gm SMN plate were streaked onto SMN plates with Nx and Km to select for those that underwent a double-crossover recombination event (no growth on Km indicates target deletion). The putative deletion mutant colonies were grown in liquid cultures under aerobic and anaerobic conditions to observe the phenotypic effect. Genomic DNA was isolated for sequencing of the flanking regions and the Gm^r^ cassette using primers within the cassette and 300 bp outside the plasmid-cloned sequences to confirm the absence of the target gene. Mutants were named with the Δ symbol and the number of the gene that they are lacking (Δ*3606*, Δ*1729*, etc.).

**Table 1:** Primer sequences for amplification of the 5’ and 3’ regions surrounding each gene of interest in *R*. *rubrum*. Sequences 0.8 kb upstream and 0.8 kb downstream of each gene were generated on the NEBuilder website.

KO Name	Primer Region	FWD primer	REV primer
Δ*1274*	5’	ccacggcgatatcggatccatatGATCAAGCGCAAGGTCATCC	tggcgaatggGGGCGGTGATAGCCCATC
	3’	cgctcactgcGACGGTCAACCTTGACCGGCG	tacgccactagtccgaggcctcgagCGCAGGCGGTGGAGGGCT
Δ*1729*	5’	ccacggcgatatcggatccaACCCATTACGGCGACATGGATATCTCG	tggcgaatggCCAGAGCGCAGCGTGCGC
	3’	cgctcactgcCGGTCTTCCTCGGGATCG	tacgccactagtccgaggccTCCGAAGGGCTGGCCAAG
Δ*2106*	5’	ccacggcgatatcggatccatatgTCAGGGTCAGCCGGGCGAG	gcgaatggGCCGCCATCGGCCGTT
	3’	cgctcactgcCGCCTTTCTCGGCGCCGT	ccatggtacccgggagctcgaattcTTGAGATCCTGGGCCTGCAGC
Δ*2553*	5’	acgcgtctgcagaagcttcgAAAGCCCGATCCGTTTCTTC	tggcgaatggGACCAGATCGGCCAGGAA
	3’	cgctcactgcCGATTTCGGCGGTCGCCG	tacgccactagtccgaggccCCTGGACGTCGCCGTGCC
Δ*2871*	5’	ccacggcgatatcggatccaTCTCGCTGATCGTCGAAG	tggcgaatggGGCCTCGTCGACCAGATC
	3’	cgctcactgcGACATCGAAGGACACCGCCTC	tacgccactagtccgaggccCCCCTTTCTGCGGGCGCT
Δ*3004*	5’	cgatccactagttctagagcCACCTTATAACCCTCCACAAACAG	tggcgaatggGAATCCGGTCACCCGCTG
	3’	cgctcactgcCGCTATGCTGGCCAAAAGCCGGGG	agacgcgtcgacgtcatatgCGGGCGCCCATCCGCCGC
Δ*3121*	5’	ccacggcgatatcggatccatatgTCACCGCCTCGCTGGCGG	tggcgaatggGAACCGCCGCCAAGCCGG
	3’	cgctcactgcCCTTGCCCCCGGCGCGTG	ccatggtacccgggagctcgaattcGCTTCTTCGGCGGGGGCG
Δ*3231*	5’	ccacggcgatatcggatccatatgCCCATCAGGGCGCTGGTG	tggcgaatggCGCCGTCTCTCCGTCCAC
	3’	cgctcactgcCCGTGCCGGGCATGGCGG	ccatggtacccgggagctcgaattcGAGGGCGGCGACCTGGGG
Δ*3606*	5’	cgatccactagttctagagcGAACGGGCGAGCAGGCGC	tggcgaatggAGGACGCGCCCCATGGATC
	3’	cgctcactgcGGTCGGTCTGACTGTCTTCCTG	ctgcagacgcgtcgacgtcaGATCGCGCCGCCATCGAG

### Lipid extraction of cells

Lipid extractions were performed on thawed cell pellets (25–40 mg) of WT and mutant strains of *R*. *rubrum* (aerobic and anaerobic). RQ_3_ internal standard (20 μmol) was added to all samples prior to extraction for a final LC-MS injection concentration of 1.25 pmol/10 μL. Cells were kept on ice and resuspended in methanol (1 mL) and H_2_O (80 μL, 18 MΩ) by vortex mixing in a 5-mL glass screw-cap centrifuge tube. Petroleum ether (0.8 mL) containing 10 μM butylated hydroxytoluene (BHT) was added to each cell suspension and mixed. Extraction mixtures were separated by centrifugation at 2000 *x g* for 5 min at 4°C (Dupont, Sorvall RT 6000B, Wilmington, Delaware). A second extraction of the aqueous layer with petroleum ether (0.8 mL, 10 μM BHT) was performed, and the ether layers were combined. The extracts were dried down using a nitrogen evaporator (Organomation, Berlin, MA) and stored at -20°C in a desiccator before resuspension. Dried lipid extracts were resuspended in LC-MS grade hexanes (80 μL) and 200 proof ethanol (320 μL). Fresh 1:200 dilutions were prepared in ethanol prior to each injection.

### Preparation of standards

LC-MS standards were prepared for the RQ_10_ quantitation in pellets, containing RQ_3_ (1.25 pmol/10 μL injection), RQ_10_ (0.8, 1.6, 3.2, 6.4, 8.0 pmol/10 μL injection), and Q_10_ (0.8, 1.6, 3.2, 6.4, 8.0 pmol/10 μL injection) in absolute ethanol. Standards were extracted using the same protocol as the pellets, except with petroleum ether containing 1 μM BHT. Dried standards were resuspended in 40 μL hexanes and 160 μL ethanol, and fresh 1:100 dilutions were prepared for each injection.

### Liquid chromatography-mass spectrometry (LC-MS) analysis of RQ and Q production

The diluted lipids extracts and standards were separated using high performance liquid chromatography (Waters Alliance 2795, Waters Corporation, Milford, MA) and quinones were quantified using a triple quadrupole mass spectrometer in positive electrospray mode (Waters Micromass Quattro Micro, Waters Corporation, Milford, MA). Chromatography was performed at 4°C using a pentafluorophenyl propyl column (Luna PFP[2], 50 by 200 mm, 3 μm, 100 Å, Phenomenex, Torrance, CA) at a flow rate of 0.5 mL/min and injection volumes of 10 μL. Quinones were eluted between 1.7 and 6.7 min by using a gradient system containing water with 0.1% formic acid (buffer A) and acetonitrile with 0.1% formic acid (buffer B). The water and acetonitrile used were liquid chromatography-mass spectrometry (LC-MS)-grade Optima (Fisher Scientific, Pittsburgh, PA), and the formic acid was >99% packaged in sealed 1-mL ampoules (Thermo-Scientific, Rockford, IL). The gradient (buffer A-buffer B) method used was as follows: 0 to 3.5 min (30:70), 3.50 to 3.75 min (30:70 to 2:98), 3.75 to 7.25 min (2:98), 7.25 to 7.5 min (2:98 to 30:70), and 7.50 to 9 min (30:70). The RQ_3_ internal standard eluted at 1.7 min, RQ_10_ at 6.6 min, and Q_10_ eluted at 6.7 min. Quantitation was accomplished using MRM of singly charged ions, and monitored for the mass transition from each quinone precursor ion ([M+H]^+^) to its respective tropylium product ion ([M]^+^). Mass Lynx V. 4.1 software was used for data acquisition and processing. Linear slopes were calculated using peak areas with a bunching parameter of 3 and three smoothing functions. The following global conditions were used for MS/MS analysis of all compounds: Capillary voltage, 3.60 kV; Source temp, 120 ^o^C; Desolvation temp, 400 ^o^C; Desolvation N_2_ gas flow, 800 L/h; and Cone N_2_ gas flow, 100 L/h. Argon gas was used as the collision gas and was obtained from the boil-off from a bulk liquid argon storage tank. Additional quinone-specific parameters are listed in Table 2.

**Table 2:** LC-MS parameters for each quinone.

MS parameter	RQ_3_	RQ_10_	Q_10_
Dwell time (ms)	200	100	100
Cone (V)	25	39	35
Collision (V)	20	30	30
Precursor mass [M+H]^+^ (*m*/*z*)	372.2	848.7	863.7
Ion product mass [M]^+^ (*m*/*z*)	182.2	182.2	197.4

Two injections of standards were analyzed to generate standard curves of RQ_10_/RQ_3_ and Q_10_/RQ_3_ response ratio versus RQ_10_/RQ_3_ and Q_10_/RQ_3_ pmol ratio, respectively. Injections of each lipid sample were performed in duplicate and the average pmol RQ_10_ and Q_10_ per mg wet pellet mass were calculated for each sample using the standard curve and corrected for recovery of RQ_3_ internal standard. This analysis was performed with three different cell pellets of WT and each mutant (semi-aerobic and anaerobic) and the pmol RQ_10_ and Q_10_ per mg wet pellet values from each sample were averaged to obtain the average total pmol RQ_10_ and Q_10_ production, per milligram of cells, for each strain. From these results, a one-tailed Student’s t-test assuming equal variances was performed, comparing each semi-aerobic mutant strain to the semi-aerobic WT production, and anaerobic mutants to anaerobic WT production. Significant differences were determined at the α < 0.05 level.

### Optimization of TaqMan assays for real-time quantitative polymerase chain reaction (RT-qPCR)

In order to compare the expression of the Rru_A3227 *(rquA)* gene in WT with the Δ*1729* and Δ*3606* mutants, several endogenous control genes were tested for use with the TaqMan Gene Expression Assay (Life Technologies, Waltham, MA). The genes tested as endogenous controls were: Rru_A0016, Rru_0454, Rru_A0917, Rru_A2015, Rru_A2882, and Rru_A3079. To determine the dynamic range of detection and validate the efficiency of the target and endogenous control assays, a relative standard curve experiment was performed over a 6-log dilution range of cDNA from WT, Δ*1729*, and Δ*3606*, in triplicate. The Rru_A3079 gene was selected for the endogenous control assay since it had similar expression under both aerobic and anaerobic growth, and was stable in all samples. The primer and probe sequences for each assay are listed in Table 3.

**Table 3:** The forward and reverse primer sequences for TaqMan assays. Primer sequences for Rru_A3227 and Rru_A3079 are listed, as well as the probe sequence.

TaqMan® Assay	Fwd primer	Rev primer	Probe
Rru_A3227	TTTGAACCCGGCCAAGAG	GCGAGAACGGTCCATAAACG	TTCTGCAGCCCGCC
Rru_A3079	GGCGGTGATGCACGTTCT	GCAGGGCCGGTATTTGGTAT	ACGAGGGTTTGGCC

### RT-qPCR on mutant *R*. *rubrum* strains

Based upon LC-MS results, total RNA from anaerobically grown WT, Δ*1729*, and Δ*3606* was isolated from cell pellets which were prepared as previously described, using the RNeasy Mini Kit (Qiagen, Palo Alto, CA) and the Quick-Start protocol for < 1x10^8^ cells. All samples underwent an on-column DNase II digestion for 15 min at 30°C during the RNA purification process, as well as a second 1-h DNase II treatment at 30°C. After the second DNase II treatment, the RNA was cleaned using the Zymo Clean and Concentrator Kit (Zymo Research, Irvine, CA). The concentration and quality of RNA (A_260/280_) was determined using a Nanodrop One UV-Vis spectrophotometer (Thermofisher Scientific, Waltham, MA). PCR with the Rru_A3231 in-gene primers (5’- TTCTGACCTTGCTGGCGATC-3’ and 3’- GGCTGATGCTGGCATCAAG-5’) was performed on each sample in order to test for genomic DNA contamination. The PCR products were visualized on a 1% agarose gel, alongside the crude RNA in order to verify that the RNA was intact. Once clean RNA was obtained, the High Capacity RNA to cDNA kit (Applied Biosystems, Waltham, MA) was used to create a cDNA library at a concentration of 120 ng/μL, the optimum concentration within the dynamic range of the TaqMan Gene Expression Assays, as determined by the standard curve experiment. A no reverse transcriptase (no RT) sample was also prepared in the cDNA reaction in order to test for the presence of genomic contamination in the RT-qPCR experiment. A standard Comparative C_T_ (ΔΔC_T_) experiment was performed with the Rru_A3227 and Rru_A3079 assays on a StepOnePlus Real-Time PCR system (Life Technologies, Waltham, MA). ROX dye was used as a passive reference and WT cDNA was used as an active reference in each experiment. Each cDNA sample and no RT control was tested in triplicate with each assay, and the average C_T_ values were generated for each biological sample with each gene target. From here, ΔC_T_ and ΔΔC_T_ values were obtained in order to determine the range of fold-change values, comparing anaerobic knock-out mutants to anaerobic WT. Relative quantitation (RQ) ranges were determined through standard propagation of error [15].

## Results

### Identification of RQ biosynthesis gene candidates

Of the 3782 *R*. *rubrum* genes for which data were obtained, 806 expressed RNA at least 3-fold higher in anaerobic versus aerobic conditions, representing ~22% of the genome (RPKM ratio greater than 2.99, S1 Table). The genomes of two purple photosynthetic bacteria were compared with *R*. *rubrum*. *R*. *ferrireducens* produces RQ and can undergo anaerobic respiration [16,17]. *R*. *sphaeroides* does not produce RQ and cannot carry out anaerobic respiration [18,19]. Of the 806 genes upregulated during anaerobic growth, 51 genes were found to be members of orthologous gene clusters shared with *R*. *ferrireducens*, but not with *R*. *sphaeroides* (S1 Table). From this abbreviated list (which includes the *rquA* gene, serving as a benchmark for involvement in RQ_10_ biosynthesis), we selected for further investigation six genes annotated with putative functions related to methyl-, amido- or aminotransferase activity, or no known function (Table 4). We selected three additional genes with putative transferase functions that were both dramatically up-regulated in anaerobic conditions and more closely related to a gene in *R*. *ferrireducens* than to *R*. *sphaeroides*, when comparing BLASTp E-values (Table 4).

**Table 4:** *R*. *rubrum* gene candidates identified from RNAseq and comparisons with RQ producer *R*. *ferrireducens* and RQ non-producer *R*. *sphaeroides*.

Gene candidate (NCBI)	RPKM Anaerobic:Aerobic	Log (E-value *Rr*:*Rf /*E-value *Rr*:*Rs)*	eggNOG orthologous gene cluster	Proposed Function
*rquA* (Rru_A3227)	8.4	-57.8	179MD	methyltransferase
Rru_A1274	24.3	-67.6	176BB	Radical SAM family protein
Rru_A2871	15.2	-18.3	—	Peptidase/amidohydrolase
Rru_A2553	13.5	-5.1	—	Ubiquinone/menaquinone biosynthesis methyltransferase
Rru_A2106	7.7	-68.4	16QF5	Hypothetical protein
Rru_A3606	7.1	-31.8	16RW4	Hypothetical protein
Rru_A1729	6.9	-35.2	16YKK	Hypothetical protein
Rru_A3004	4.5	-73.4	16Q21	Class I and Class II aminotransferases
Rru_A3121	3.2	-170.0	—	Asparagine synthase
Rru_A3231	3.0	-57.0	1748D	Isoprenoid biosynthesis protein with amidotransferase-like domain

RNA expression differences in anaerobic and aerobic conditions are shown as ratios of RPKM measured in each condition. Comparisons in similarity between each *R*. *rubrum (Rr)* gene and its closest relative in *R*. *ferrireducens (Rf)* and *R*. *sphaeroides (Rs)* are shown as ratios of BLASTp E-values. When *R*. *rubrum* genes are members of orthologous gene clusters shared with *R*. *ferrireducens* but not with *R*. *sphaeroides*, the eggNOG cluster number is shown. Proposed functions are the annotations on each gene in the *R*. *rubrum* ATCC 11170 genome from NCBI. The complete set of RNAseq data and eggNOG analysis may be found in the S1 Table.

**Fig 2:**
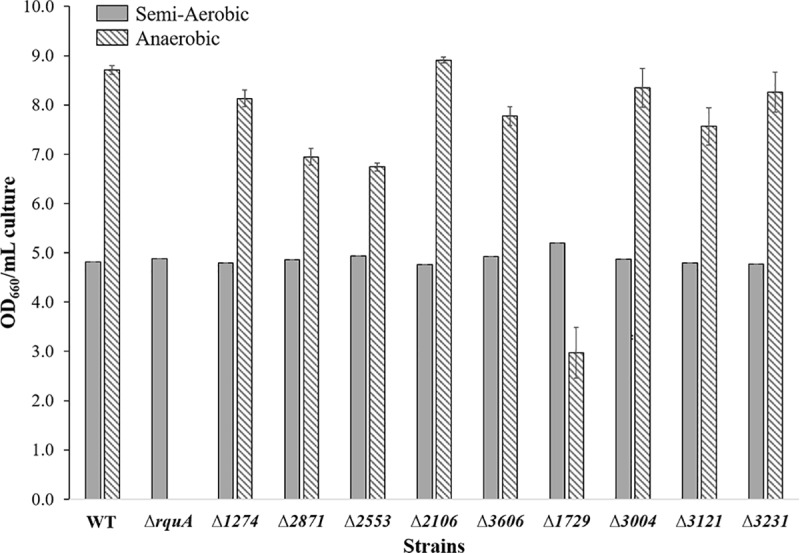
OD_660_ values for 1 mL cultures of *R*. *rubrum* mutant strains compared to WT. The striped bars represent OD_660_ values reached under anaerobic growth conditions and the grey bars indicate OD_660_ values after reaching semi-aerobic growth conditions. All anaerobic cultures were grown five days at 30°C, and semi-aerobic cultures were harvested after 2.5 days. No growth occurred in Δ*rquA* under anaerobic conditions.

Each deleted gene region was amplified by PCR using primers outside of the flanking sequences cloned in the knock-out plasmids, and Sanger sequencing verified that the coding regions of each gene had been deleted and replaced by the Gm^r^ gene. While none of the knockouts halted the anaerobic growth of *R*. *rubrum*, as in the Δ*rquA* mutant, it was observed that compared to WT, anaerobic Δ*1729* growth was slower, and significantly lower cell density was obtained after 5 days of growth (OD_660_ 2.9 ± 0.8) (Fig 2). The semi-aerobic cultures grew to a similar OD_660_ in all strains (Fig 2).

### LC-MS quantitation of RQ and Q in knock-out mutants

**Fig 3:**
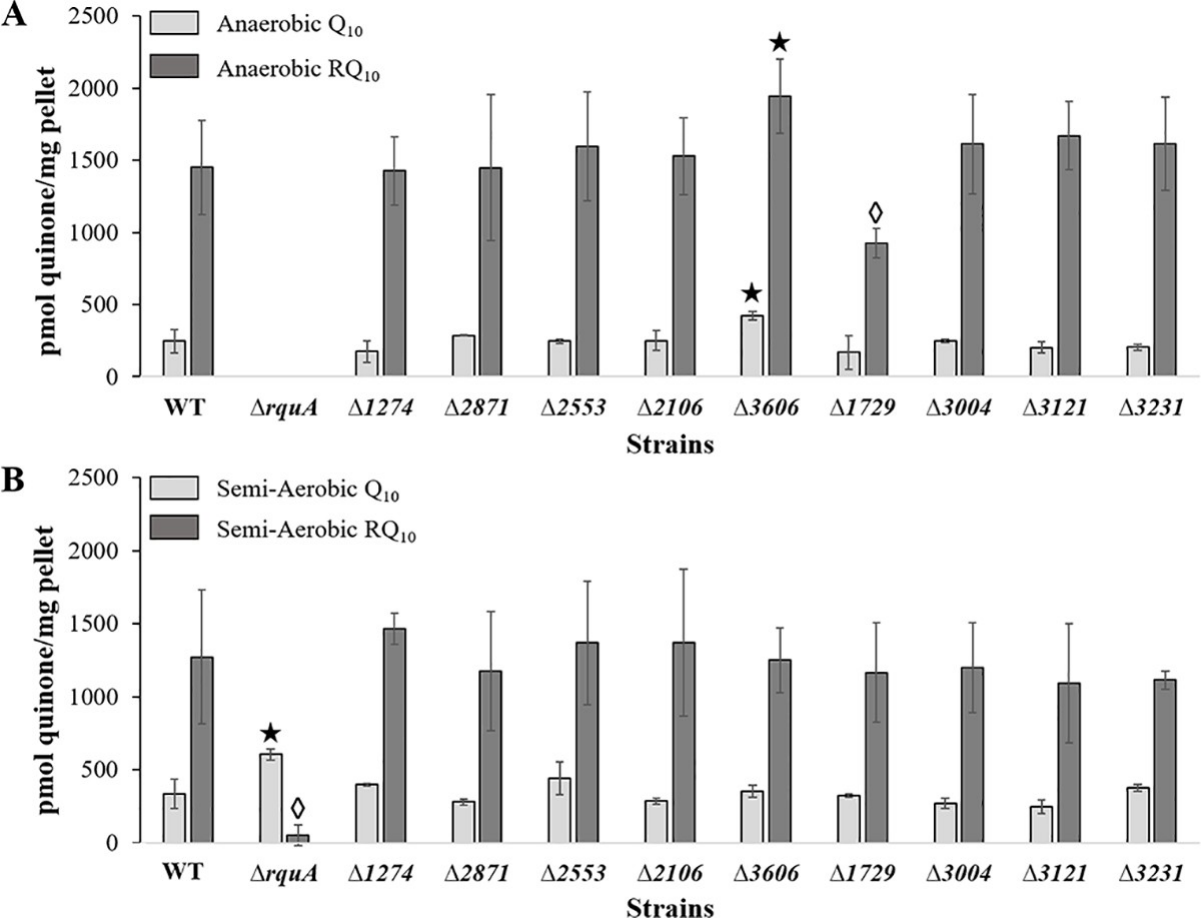
RQ_10_ and Q_10_ production (pmol/mg wet pellet) in *R*. *rubrum* mutant strains from LC-MS analysis. A. Anaerobic growth conditions with RQ_10_ production designated with dark grey bars and Q_10_ with light grey bars. No growth of Δ*rquA* occurred under anaerobic conditions. B. Semi-aerobic growth conditions with dark grey bars for RQ_10_ and light grey bars for Q_10_ production. Diamonds (◊) indicate significant decreases and stars (★) indicate significant increases in comparison to WT at the α < 0.05 level.

The results from LC-MS quantitation of RQ_10_ and Q_10_ produced under anaerobic growth conditions in the WT and mutant strains are shown in Fig 3A. The Student’s t-test showed that anaerobic Δ*1729* produced significantly less RQ_10_ than anaerobic WT (p = 0.028), as well as reduced levels of Q_10_. Anaerobic Δ*3606* produced more RQ_10_ than anaerobic WT with a weak significance at the α < 0.05 level (p = 0.054). The level of Q_10_ in the anaerobic Δ*3606* sample was significantly increased compared to WT (p = 0.012). The quinone levels from the semi-aerobically grown bacteria are listed in Fig 3B. The semi-aerobic Δ*rquA* mutant produced significantly less RQ_10_ as compared to semi-aerobic WT, as expected (p = 0.003), and significantly more Q_10_ (p = 0.006).

### RT-qPCR analysis of gene expression in knock-out candidates

**Fig 4:**
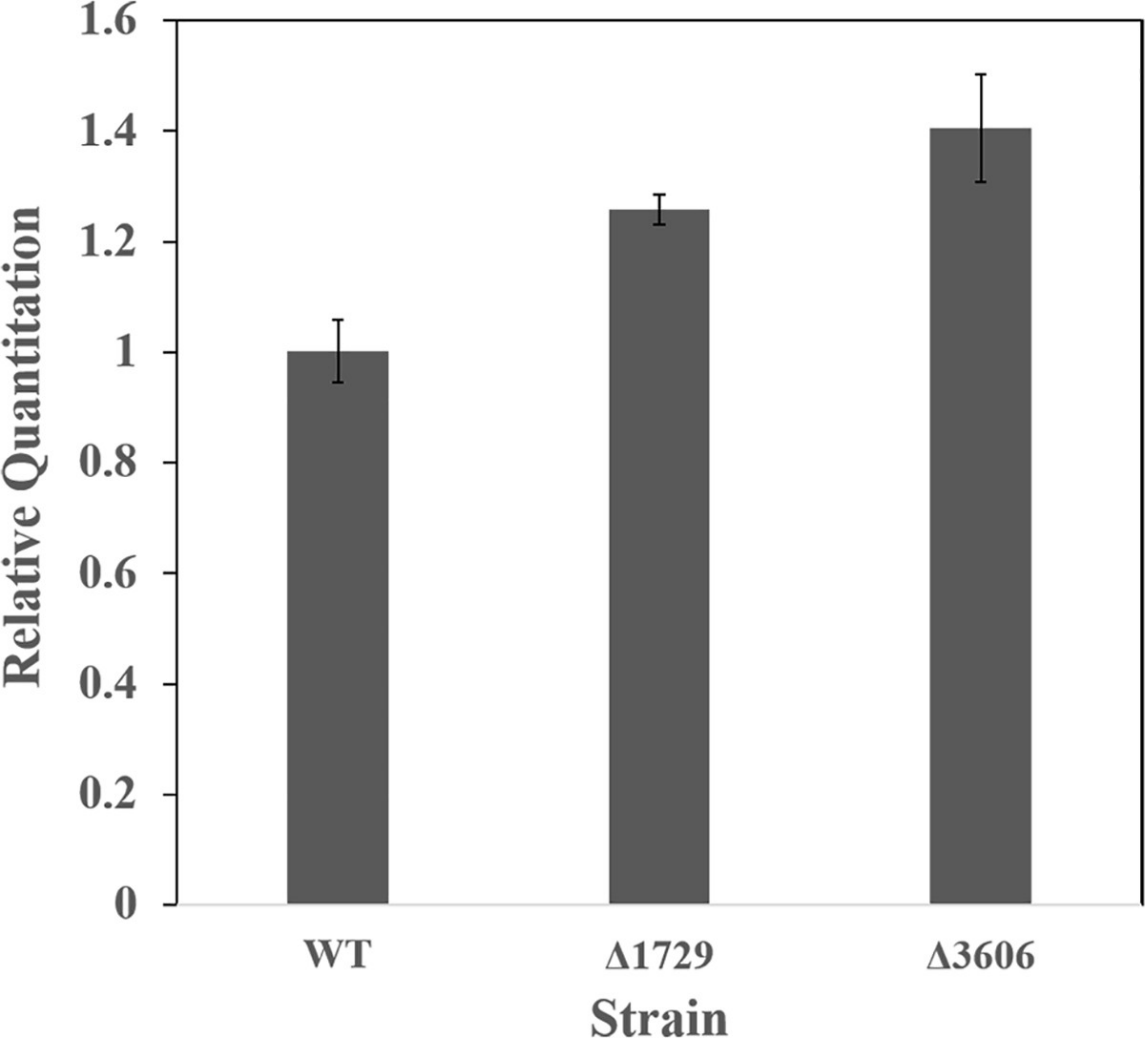
RT-qPCR Relative Quantitation (RQ) of anaerobic Δ*1729* and Δ*3606* RNA compared to WT. There was a 1.2-fold increase in *rquA* expression in the Δ*1729* mutant, and a 1.4-fold increase in Δ*3606*.

The LC-MS results prompted further investigation into the *rquA* gene expression in the Δ*1729* and Δ*3606* strains. The standard curve experiment for optimization of the TaqMan assays determined that the target (Rru_A3227) and endogenous control (Rru_A3079) assays gave slopes and amplification efficiencies were approximately equal. The Rru_A3227 assay had 109% efficiency with a slope of -3.12 and R^2^ = 0.97. The Rru_A3079 assay had 111% efficiency with a slope of -3.09 and R^2^ = 0.99. Because of these similarities, RT-qPCR was performed on RNA isolated from Δ*3606* and Δ*1729* mutants using the ΔΔC_T_ method for analysis. The RT-qPCR results showed that, under anaerobic growth, the Δ*3606* mutant had 1.4-fold increase in *rquA* gene expression as compared to WT, and the Δ*1729* mutant had a 1.2-fold increase (Fig 4). The differences in *rquA* expression between the mutants and WT are quite small (<2-fold increase), and do not demonstrate a strong correlation between RQ levels and *rquA* expression.

## Discussion

To search for additional genes that may be involved in RQ biosynthesis in *R*. *rubrum*, we used a candidate gene approach. Candidate genes were deleted from *R*. *rubrum*, and then we assayed for effects on anaerobic growth and the quantity of RQ and Q in the mutant *R*. *rubrum* strains. We reasoned that genes involved in RQ biosynthesis would be expressed at a higher level during anaerobic growth, when RQ production is essential, than during aerobic growth, when RQ is not necessary. We also anticipated that such genes would be more closely related to genes in bacteria that produce RQ than to genes in bacteria that do not produce RQ. We further reasoned that since RQ contains an amino group where Q has a methoxy group, genes involved in RQ synthesis might encode proteins with transferase activities, or perhaps proteins with no known function. We chose to delete the Rru_A2553 gene since it was annotated in the NCBI genome database as a ubiquinone/menaquinone methyltransferase. We predicted that if Q levels were reduced in this mutant, a similar effect would be observed on RQ levels, since Q has been proposed to be a precursor to RQ [11]. It is known that in *Saccharomyces cerevisiae*, Q biosynthesis requires a complex of at least eleven proteins [20], and therefore we previously hypothesized that RquA may similarly function in conjunction with other enzymes, such as an amido- or aminotransferase, as part of a multicomponent complex for RQ biosynthesis [12]. The deletion of genes required for a biosynthetic complex would be predicted to affect the synthesis of RQ and possibly the expression of *rquA*.

The LC-MS results presented indicate that *rquA* is more essential for RQ production in *R*. *rubrum* than any of the new candidate genes tested. However, some of the gene deletions in this study affected the levels of Q in anaerobically grown *R*. *rubrum*, which were in direct correlation to RQ levels. For example, deletion of Rru_A3606 resulted in a higher production of both Q and RQ under anaerobic conditions, while under semi-aerobic conditions, the amounts were consistent with the WT control. These data suggest the Rru_A3606 gene product could be involved in modulating Q synthesis or *rquA* expression. In contrast, reduced amounts of both Q and RQ were observed in Rru_A1729 in the absence of oxygen, while little variance was observed under semi-aerobic conditions. These results indicate that Rru_A1729 may be required to achieve growth equivalent to the WT strain in anaerobic conditions, and suggests that the anaerobic growth problem could be a result of lower quinone levels. Both examples support the observation that Q is a required biosynthetic intermediate to RQ [11], and specifically, Rru_A3606 and Rru_A1729 gene deletions may be affecting the regulation of Q synthesis under anaerobic conditions. We did not observe significant differences in Q or RQ levels in the Δ*2553* mutant under either growth condition. Even though Rru_A2553 is annotated as a ubiquinone/menaquinone methyltransferase, there are alternate methyltransferases present in the *R*. *rubrum* genome with higher sequence similarity to *coq3/ubiG* (Rru_A0742) and *coq5/ubiE* (Rru_A3798), the known methyltransferases used in Q biosynthesis in yeast and *E*. *coli*. Finally, the level of Q in the Δ*rquA* mutant was significantly increased compared to WT under semi-aerobic conditions, perhaps because it cannot serve as intermediate to RQ in the absence of RquA.

The effect on *rquA* RNA levels in the anaerobically grown mutants was less significant. For example, there was only a 1.4-fold increase of *rquA* gene expression in Δ*3606*, indicating that *rquA* RNA quantity is not closely linked to RQ levels. It is instead possible that Rru_A3606 inhibits the flux of Q precursors, or affects the stability of Q or RQ after they are produced. We cannot rule out the possibility that the Δ*3606* phenotype results from a polar effect on downstream genes in a shared operon. Additionally, the Δ*1729* mutant is not reducing RQ production by inhibiting *rquA* expression, as a 1.2 fold increase was observed. Normal Rru_A1729 activity could be necessary for adequate production of RQ precursors, improving activity of upstream enzymes in the pathway, or inhibiting enzymes that degrade RQ. For both these genes, sequence homology provides no clear hints concerning their functional roles.

Since it has been observed that RQ is required for photooxidase activity during photoheterotrophic growth of *R*. *rubrum* [21], a potential photosynthetic role for RquA may exist. Careful studies of *R*. *rubrum* growth and redox balance under microaerophilic conditions in the dark have established that the redox state of the quinone pool is crucial in regulation of gene expression, and in particular the observed induction of photosynthetic membrane production [22,23]. Under these conditions, *R*. *rubrum* is also metabolically poised between oxidative and reductive modes of operation of TCA enzymes. It remains to be seen how RquA-generated RQ levels, presumably promoting reduction of fumarate, affect QH_2_/Q ratios and in turn on their downstream effects.

A recent paper by Stairs et al. [13] has provided important additional information that aids interpretation of the results we report here. As mentioned earlier, the essential role of RquA could potentially be explained by its necessary inclusion in a multienzyme RQ-synthesizing complex that shares components with an analogous complex synthesizing Q, as was shown for the yeast Coq5 *C*-methyltransferase [24]. However, Stairs et al. find that many eukaryotic organisms possessing an *rquA* ortholog do not possess the components for Q biosynthesis. Hence RquA could not be functioning in a complex including such components. Additionally, these authors used phylogenetic profiling in order to identify other enzymes with the same sparse distribution among bacterial and simple eukaryotic organisms as RquA (similar to what we report here for the two comparison bacterial genomes *R*. *sphaeroides* and *R*. *ferrireducens*, without the benefit of *R*. *rubrum* transcriptome data, but broader in phylogenetic scope). This search did not reveal any gene co-occurring with *rquA*. The implication is that either RquA alone is sufficient for conversion of Q to RQ, or that this conversion requires the activity of RquA along with one or more additional enzymatic functions provided fortuitously by more broadly distributed gene product(s) that contribute as well to other metabolic transformations. As Stairs et al. [13] conclude, in either case, the lateral transfer of the *rquA* gene could confer on perhaps many recipient organisms the ability to convert Q to RQ. This scenario would also explain why none of the *R*. *rubrum* genes investigated here by knockouts were, like *rquA*, found to be essential for RQ production.

Our results have eliminated nine genes candidates as being essential for RQ biosynthesis in *R*. *rubrum*. We propose that RquA may be acting alone in the conversion of Q to RQ. RquA is predicted to be a SAM-dependent methyltransferase; however, it is missing several key residues in the SAM-binding motif [12] that are present in orthologs for which methyltransferase activity has been observed, such as UbiG and Coq3 [25]. There have been several recent examples reported that demonstrate an alternate function of enzymes that have a conserved SAM-binding methyltransferase fold [26–28]. In both the biosynthesis of the polyketide antibiotic β-rhodomycin [28], and the biosynthesis of the natural product leporin B [26], SAM acts as an electrostatic catalyst and not as a methyl donor. It is therefore possible that RquA is acting as a transaminase with SAM as a cofactor to stabilize a negatively charged intermediate produced during a direct addition of ammonia to Q. Future work will investigate *in vitro* assays of RquA with Q as a substrate, to determine if RquA is the only additional protein required for RQ biosynthesis in *R*. *rubrum*.

## Supporting information

S1 Table The 3782 annotated genes in the *R*. *rubrum* genome are sorted using three different criteria. Genes were screened using: 1) the RPKM ratio of anaerobic:aerobic mRNA transcripts (RPKM ratio greater than 2.99); 2) closest similarity to *R*. *ferrireducens*, a RQ producing species versus *R*. *sphaeroides*, a non RQ-producing species (logRat_RrRf_to_RrRs below -5); and 3) by presence of eggNOG orthologous clusters in *R*. *ferrireducens*, but not *R*. *sphaeroides* (eggNOG_Rr-Rf-NotRs). Yellow highlighting indicates that a criterion was met. There were 45 genes that met all three criteria, and the seven highlighted in orange were selected for deletion in this study (which includes *rquA*, Rru_A3227). Three additional genes were selected for this study that met the first two criteria are highlighted in blue.
(XLSX)
